# Supplementary material for: Pathogenic PSEN1 Glu184Gly Mutation in a Family from Thailand with Probable Autosomal Dominant Early Onset Alzheimer’s Disease
Source: Diagnostics (Basel). 2020 Mar 1;10(3):135. doi: 10.3390/diagnostics10030135 (PMC7151116; doi:10.3390/diagnostics10030135)
Supplement: Supplementary file 1 [file diagnostics-10-00135-s001.pdf]

diagnostics

MDPI

Supplementary table 1. : List of variants, found by the targeted NGS analysis.

Chromosome	Gene	Location	Mutation	RsID	SIFT		Polyphen2		1000Genomes	ExAC
1	CR1	c.4973A>G	p.H1658R	rs2274567	0.897	T	0.168	B	2.23E-01	2.58E-01
		c.6178A>T	p.T2060S	rs4844609	0.804	T	0	B	0.9922	9.85E-01
		c.6193A>G	p.I2065V	rs6691117	1	T	0	B	0.38919	3.27E-01
		c.6830C>G	p.P2277R	rs3811381	0.446	T	0.032	B	0,0.1964	2.40E-01
		c.7255A>G	p.T2419A	rs2296160	0.987	T	0.001	B	0.78846	8.08E-01
2	ALS2	c.1102G>A	p.V368M	rs3219156	0.191	T	0.009	B	0.8983	9.10E-01
5	CSF1R	c.1085A>G	p.H362R	rs10079250	0.061	T	0.157	B	0.1575	4.89E-02
10	OPTN	c.293T>A	p.M98K	rs11258194	0.925	T	0.004	B	0.0677	4.49E-02
		c.964G>A	p.E322K	rs523747	1	T	0	B	9.91E-01	9.97E-01
	CTNNA3	c.1787G>A	p.S596N	rs4548513	1	T	0.001	B	0.4725	4.12E-01
11	BACE1	c.1385A>G	p.N462S	rs539765	1	T	0	B	1	1.00E+00
	SORL1	c.3220G>C	p.E1074Q	rs1699107	0	T	0	B	0.9885	9.95E-01
		c.5899A>G	p.I1967V	rs1792120	1	T	0.003	B	0.9853	9.95E-01
12	LRP6	c.3184G>A	p.V1062I	rs2302685	1	T	0	B	0.8777	8.47E-01
	LRRK2	c.149A>G	p.H50N	rs2256408	1	T	0	B	0.9739	9.91E-01
		c.4939T>A	p.1647T	rs11564148	0.953	T	0	B	0.2751	2.96E-01
		c.7190T>C	p.M2397T	rs3761863	0.466	T	0	B	0.54945	6.17E-01
14	PSEN1	c.551A>G	p.E184G	rs1566641934	0.005	D	0.733	P	NA	NA
15	SPG11	c.833A>G	p.N278S	rs75309308	0.487	T	0.01	B	0.0201	7.76E-03
17	MAPT	c.1321T>C	p.Y441H	rs2258689	0.978	T	0.001	B	3.24E-01	2.75E-01
19	ABCA7	c.955A>G	p.T319A	rs3752232	0.882	T	0.001	B	0.1011	5.99E-02
		c.1184A>G	p.H395R	rs3764647	1	T	0.002	B	0.09706	5.76E-02
		c.1185C>G	p.H395Q	rs3764647	0.704	T	0.007	B	0.108826	6.30E-02
		c.1388G>A	p.R463H	rs3752233	0.254	T	0.796	P	0.0590	4.70E-02
		c.2153A>C	p.N718T	rs3752239	0.239	T	0.182	B	0.0581	4.58E-02
		c.4580G>C	p.G1527A	rs3752246	0.877	T	0	B	0.8237	8.38E-01
		c.5057A>G	p.Q1686R	rs4147918	0.234	T	0.004	B	0.05586	4.78E-02
	NOTCH3	c.6668C>T	p.A2223V	rs1044009	0.175	T	0.003	B	0.6309	6.94E-01
	CD33	c.205A>G	p.R69G	rs2455069	0.335	T	0.003	B	0.3489	3.58E-01
20	PRNP	c.385A>G	p.M129V	rs1799990	0.024	D	0.012	B	0.2637	3.07E-01
